# YFDM: YOLO for detecting Morse code

**DOI:** 10.1038/s41598-023-48030-7

**Published:** 2023-11-23

**Authors:** Zhenhua Wei, Zijun Li, Siming Han

**Affiliations:** Academy of Operational Support Rocket Force Engineering University, Xi’an, 710025 China

**Keywords:** Computer science, Electrical and electronic engineering

## Abstract

With the increasing complexity of the shortwave communication environment, the efficiency and accuracy of the manual detection of Morse code no longer meet actual needs. Therefore, this paper proposes a Morse code detection algorithm called YFDM. For the time–frequency image of the received signal, a combination module of deformable convolution and C3 is used to enhance the backbone network’s attention to the abstract semantics and location information of Morse code. GSConv and VOV-GSCSP modules are used to build a lightweight neck network. Finally, the confidence propagation cluster (CP-Cluster) algorithm is used to filter the detection frame. In an ablation experiment, the parameters and giga floating-point operations per second (GFLOPs) of YFDM were 5.961 M and 9.74 G, respectively, 15.11% and 38.9% less than those of YOLOv5. Moreover, when WIoUv1 was used as the loss function of the bounding box, the AP0.5:0.95 and frames per second (FPS) values of the algorithm reached the highest values, 0.68 and 72.4. The experimental results indicate that the algorithm can effectively reduce the weight of the model while ensuring the detection accuracy and inference speed.

## Introduction

Morse communication is widely used in military, maritime, and other special fields because of its fast connection, long transmission distance, and strong anti-interference ability. Earlier developed Morse code detection methods require the prior information of the signal to be detected^1–6^ and are sensitive to noise interference^7–9^, limiting their application scope. As time–frequency analysis gradually became the mainstream preprocessing method for Morse code feature extraction, researchers began to use decision trees, support vector machines, and other classifiers to distinguish various signals in time–frequency images, and gradually developed a class of detection methods based on fast narrowband filtering and classifiers^10–15^. Although the introduction of machine learning improves the robustness and practicability of time–frequency-analysis-based detection methods, problems remain. First, unreasonable noise floor estimation and threshold setting can easily cause missing detection in signal filtering. Second, the geometric deformation of Morse code due to frequency offset and other factors increases the possibility of signal misjudgment.

With the development of deep learning technology, Yuan et al.^16^ introduced a convolutional neural network into a Morse code detection task to achieve detection accuracy of more than 97%. Recently, researchers used a single-shot multi-box detector (SSD)^17^ to detect multiple types of signals in the time–frequency diagram. However, Zha et al.^18^ found that it was difficult for SSD to detect complete long and narrow signals. Therefore, Li et al.^19^ proposed a detector based on signal centerline modeling, which achieved better average detection accuracy and processing speed than Faster-RCNN^20^ and SSD detectors. Detectors based on YOLO^21^ also showed great potential in the task of signal detection and classification. Researchers^22^ also proposed a new method of IoT signal detection and classification based on YOLO. Vagollari et al.^23^ successfully detected RF signals with different time and frequency spans using YOLOv3. YOLO detectors were also used to detect constellation diagrams of different signals^24^.

We use the signal time–frequency image as the processing object and implement the fast detection of multiple Morse code by refining the specific object detection algorithm, in light of the successful application of object detection algorithms in the detection of communication signals. Before designing the model of the automatic Morse code detection algorithm, the following factors are also taken into account:

1. Considering the real-time nature of Morse communication, the detection algorithm model should have a fast processing speed, so a one-stage target detection model is prioritized.
2. Computational resources are more constrained in motorized communication scenarios, and pertinent hardware devices typically need to manage several parallel tasks. In order to ensure that the algorithm model has good detection accuracy and processing speed while still meeting actual deployment requirements, it should be made lighter in order to minimize the amount of hardware computational resources it occupies. This way, the model can still function well even when faced with computational resource limitations.
3. When the signal-to-noise ratio is low, Morse code may exhibit various changes in characteristics such as speed deviation, frequency shift, point, and scratch in the signal time–frequency image. Consequently, it is imperative to augment the network's capacity to extract Morse code features and elevate the network's capability to identify unstable and low-quality Morse codes.
4. There is a need to introduce a more suitable candidate box selection algorithm because, while the traditional NMS algorithm retains the candidate box with the highest confidence score among all categories during the candidate box selection stage, it is difficult to guarantee that this detection box is the best and the method is more sensitive to the intersection and concurrency ratio threshold setting.

The YFDM (YOLO for Detecting Morse) algorithm model is proposed in this paper to address the aforementioned motivation. Its main work is summarized as follows:

1. The backbone network uses deformable convolution^25^ and C3 combination modules to enhance the representation ability of Morse code's geometric characteristics and make up for the defect of fuzzy location information in the deep network.
2. The neck network uses a combination module of GSConv and VOV-GSCSP to simultaneously ensure the detection accuracy and reduce the amount of model parameters and computational complexity^26^.
3. The confidence propagation cluster^27^ algorithm is used to obtain the interaction information between candidate frames and complete the screening to obtain high-quality detection frames.
4. The influence of different IoU bounding-box loss functions on the Morse code detection performance is compared and analyzed, providing reference and data support for IoU loss selection for related tasks.
5. A Morse code time–frequency graph dataset is prepared, imitating a real Morse communication scene.

In the “YFDM detection model” section, the YFDM algorithm model and processing flow are introduced in detail. In the “Experiment” section, we present experimental verification of the proposed method and demonstrate the detection effect. The “Conclusion” section provides the conclusion, which summarizes the main work and experimental conclusions of this paper and puts forward prospects for future work.

## YFDM detection model

### Model overview

**Figure 1:**
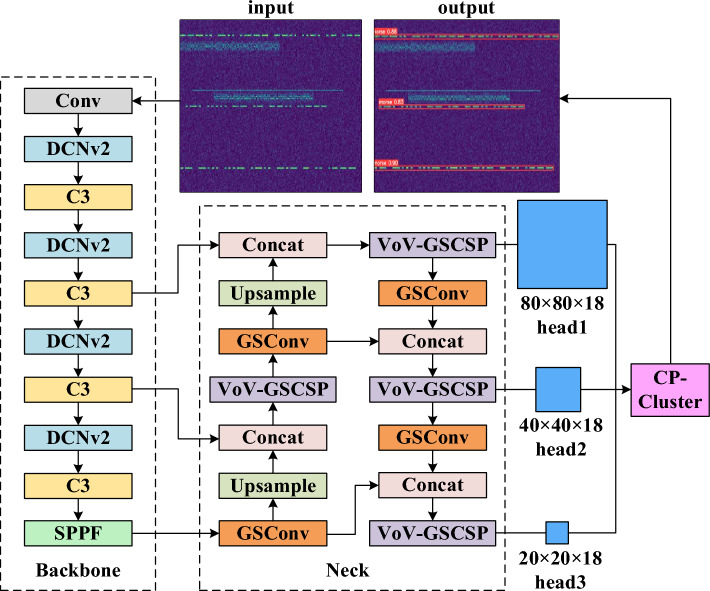
Schematic diagram of the YFDM model frame.

The overall framework of the YFDM detection model is shown in Fig. 1, which corresponds to the four stages of feature extraction, feature fusion, detection frame reasoning, and post-processing. The parameter settings of each layer of the network model are shown in Table 1.

**Table 1:** Parameter setting and output feature drawing size of each layer (settings: [out channels, kernel size, stride, padding]; feature maps: [w, h, out channels]).

	Layer	Settings	Feature maps		Layer	Setting	Feature maps
1	Conv	64, 6, 2, 2	320,320,64	14	VoVGSCSP × 3	512, /, /, /	40,40,512
2	DCNv2	128, 3, 2, /	160,160,128	15	GSConv	256, 1, 1, /	40,40,256
3	C3	128, /, /, /	160,160,128	16	Upsample	–	80,80,256
4	DCNv2	256, 3, 2, /	[80,80,256	17	Concat	–	80,80,256
5	C3	256, /, /, /	80,80,256	18	VoVGSCSP × 3	256, /, /, /	80,80,256
6	DCNv2	512, 3, 2, /	40,40,512	19	GSConv	256, 3, 2, /	40,40,256
7	C3	512, /, /, /	40,40,512	20	Concat	–	40,40,256
8	DCNv2	1024, 3, 2, /	20,20,1024	21	VoVGSCSP × 3	512, /, /, /	40,40,512
9	C3	1024, /, /, /	20,20,1024	22	GSConv	512, 3, 2, /	20,20,512
10	SPPF	1024, 5, /, /	20,20,1024	23	Concat	–	20,20,512
11	GSConv	512, 1, 1, /	20,20,512	24	VoVGSCSP × 3	1024, /, /, /	20,20,1024
12	Upsample	–	40,40,512	25	Detect	–	–
13	Concat	–	40,40,512				

A time–frequency image of a short-wave signal with a bandwidth of 2 MHz is used as the input to the backbone network. The first convolutional layer downsamples the input image, reducing its width and height dimensions to 320 and increasing the number of channels to 64. The DCNv2 module then uses the offsets of the sampling points to modify the convolutional kernel's sensory field and enhance the network's focus on the Morse code region by using the weight coefficients, all the while suppressing the extraction of insufficient information. Three output feature maps with tensor shapes of 80 × 80 × 256, 40 × 40 × 512, and 20 × 20 × 1024 are sent to the neck network's left branch from the backbone network. The up-sampled feature maps are then fused with the coarser-grained feature maps in the backbone network using the concat operation to obtain semantic features at different levels. To aid in the network's improved localization of the Morse code region, the right branch feeds shallow location data into the convolutional layer's finer-grained feature maps. Among these, the newly added GSConv and VoV-GSCSP modules successfully lower the computational complexity and number of model parameters. After receiving the output feature maps at three different scales from the neck network, the detection head network outputs the coordinates of all candidate bounding boxes according to different anchor box sizes, and finally uses the CP-Cluster algorithm to perform the final screening of these candidate boxes. After the processing of the above process, the regions in the time–frequency image of the input signal that may be Morse code will be boxed out, thus realizing the detection of Morse code. The following sections elaborate on the processing flow of each stage of the proposed model. The detection head network outputs the coordinates of all candidate bounding boxes according to various anchor box sizes after receiving the output feature maps at three different scales from the neck network. The CP-Cluster algorithm is then used to carry out the final screening of these candidate boxes. Following the aforementioned procedure, the areas of the input signal's time–frequency image that might contain Morse code will be boxed out, which allows the detection of Morse code. The processing flow of each stage of the proposed model is explained in more detail in the sections that follow.

### Backbone network based on DCN and C3 module

The initial input of the detection model is the RGB signal time–frequency map, with a size of 640 × 640. The image needs to be first down-sampled through a convolution layer with a size of 6 × 6, and the feature map after down-sampling is input to the feature extraction backbone network. The network uses multi-layer DCNv2 and C3 combination modules to extract semantic information from feature maps of different scales, and it finally uses the SPPF module to fuse feature maps with different receptive fields.

Since Morse code may cause irregular changes in geometric features owing to frequency offset, code length deviation, or scale scaling, a conventional convolution kernel can only learn geometric feature changes of Morse code by expanding enough training data owing to the lack of an internal mechanism for processing image geometric feature transformation, which is not friendly to the Morse signal detection task under small sample conditions. To solve this problem, DCNv2 uses the offset and weight coefficient of the convolution kernel sampling points to enhance the attention to useful features and suppress the interference of redundant features. Its calculation formula is as follows:

1 $$y{\text{(P}}_{{0}} {) = }\sum\limits_{{{\text{P}}_{n} \in R}} {w({\text{P}}_{n} )} \cdot x({\text{P}}_{0} + {\text{P}}_{n} + \Delta {\text{P}}_{n} )$$

where *R* is the receptive field size of the convolution kernel, $w$ is the weight of each coordinate point, and ${\text{P}}_{0}$ is the position where each point in the output feature map $y$ corresponds to the center of the convolution kernel and maps to the input feature map. ${\text{P}}_{n}$ is the relative coordinates of each position of the convolution kernel corresponding to ${\text{P}}_{0}$, and $\Delta {\text{P}}_{n}$ represents the offset of the coordinates of each position. In the model training process, the DCNv2 module promotes the convolutional sampling points to adjust their distribution shape following the geometric changes in Morse code by minimizing the error between the predicted offset and the actual offset, and it improves the convolutional network's attention to the semantic information and location information of Morse code by updating the weight coefficient of the sampling points so that the receptive field of the output feature map is more easily concentrated in the Morse code area. To some extent, the DCNv2 module can compensate for the positioning error caused by the fuzzy position information of Morse code owing to the deepening of the network, and it can help map the features of the deep more accurately to the corresponding position of Morse code in the original image. In addition, the DCNv2 module significantly reduces the computational complexity at the cost of slightly increasing the number of model parameters.

**Figure 2:**
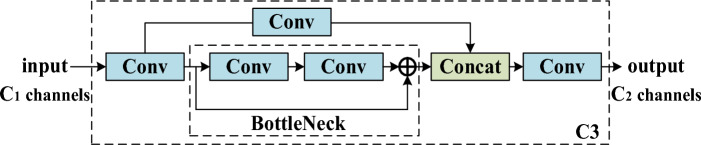
C3 module.

C3 divides the output of DCNv2 into two branches for processing. One branch enters BottleNeck to further extract deep abstract features, and the other branch uses a residual structure to transfer shallow details. Its structure is shown in Fig. 2. The C3 module appropriately increases the depth and receptive field of the network, which helps extract more fine-grained features, making Morse-code-related feature semantics more representational. The residual structure also alleviates the gradient extinction caused by the deepening of the network and reduces the computing bottleneck.

For ease of explanation, the combined feature extraction module of DCNv2 and C3 is abbreviated as DC_*i*_, where *i* represents the number of layers of the combined module connected in a series, and 320/2^*i*^ represents the size of the *i*-th layer output feature map after being connected in series. Finally, three new output feature maps (*F*_2_, *F*_3_, and *F*_4_) can be obtained, where *F*_4_ is the feature map obtained from the output of the four layer combination module after feature fusion through the SPPF layer.

2 $$F_{2} = DC_{2} (F_{1} ),F_{3} = DC_{3} (F_{1} ),F_{4} = {\text{SPPF}} (DC_{4} (F_{1} ))$$

### Neck network based on GSConv and VoV-GSCSP

**Figure 3:**
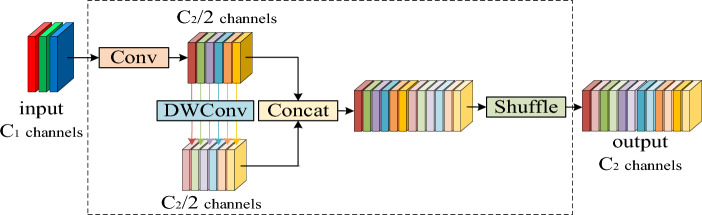
GSConv module.

The proposed model uses the structure of the feature pyramid network (FPN)^28^ and path aggregation network (PAN)^29^ in the neck network to conduct multi-scale fusion of feature maps. The FPN structure can transfer the deep feature information of Morse code to the shallow layer to enhance the multi-scale semantic expression ability, and the PAN structure can transfer the position information in the shallow feature to the deep layer to enhance its positioning ability. To reduce the amount of model parameters and computational complexity, we employ a lightweight convolution module named GSConv in this structure, as shown in Fig. 3.

GSConv uses a shuffle strategy to infiltrate the generated information of standard convolution into the generated information of depth-wise separable convolution (DSC)^30^; to some extent, it retains the hidden connection between channels, solves the defect that DSC cannot interact with local feature information between different channels, and enhances the nonlinear expression ability of the network. The calculation complexity comparison between GSConv and Standard Convolution (SC) is shown in Eq. (3):

3 $$\frac{{O_{{{\text{GSConv}}}} }}{{O_{{{\text{SC}}}} }} = \frac{{W \cdot H \cdot K_{1} \cdot K_{2} \cdot (C_{1} + 1) \cdot \frac{{C_{2} }}{2}}}{{W \cdot H \cdot K_{1} \cdot K_{2} \cdot C_{1} \cdot C_{2} }} = \frac{1}{2} + \frac{1}{{2C_{1} }}$$

where *W* is the width of the output feature map, *H* is the height of the output feature map, *K*_1_ and *K*_2_ are the sizes of the convolutional kernel, *C*_1_ is the number of channels in the convolutional kernel, and *C*_2_ is the number of channels in the output feature map. When *C*_1_ is larger, the computational complexity of GSConv is closer to 50% of SC. Since the feature map reaching the neck network has a larger channel size and a smaller height and width size, and most of the redundant information has been filtered, GSConv can maximize its lightweight advantages. Although GSConv is also used for lightweight backbone networks, the premature stacking of GSConv will significantly increase the network depth, resulting in large positioning errors in the characteristic map of the incoming neck network. Therefore, this paper only uses the GSConv module in the neck network.

**Figure 4:**
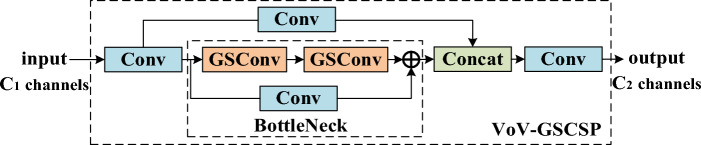
VoV-GSCSP module.

VOV-GSCSP features a GSConv-based cross-phase local network module through the single aggregation method, as shown in Fig. 4.

Its role is consistent with C3, but it has less computational complexity. In the BottleNeck section, VOV-GSCSP uses GSConv to replace the SC of C3 and places a convolution layer on the shortcut path, which enriches the gradient combination information and helps overcome the problem that the model's positioning ability declines owing to network deepening. For convenience of explanation, GSConv, VOV-GSCSP, and Upsample modules are abbreviated as GSC, VoV and Up, respectively, and the output characteristic diagram after each Concat can be obtained as in Eq. (4).

4 $$\left\{ {\begin{array}{*{20}l} {FC_{1} = {\text{Concat}} \left[ {F_{3} ,{\text{Up}} ({\text{GSC}} (F_{4} ))} \right]} \hfill \\ {FC_{2} = {\text{Concat}} \left[ {F_{2} ,{\text{Up}} ({\text{GSC}} ({\text{VoV}} (FC_{1} )))} \right]} \hfill \\ {FC_{3} = {\text{Concat}} \left[ {{\text{GSC}}({\text{VoV}} (FC_{2} )),{\text{GSC}} ({\text{VoV}} (FC_{1} ))} \right]} \hfill \\ {FC_{4} = {\text{Concat}} \left[ {{\text{GSC}}(F_{4} ),{\text{GSC}} ({\text{VoV}} (FC_{3} ))} \right]} \hfill \\ \end{array} } \right.$$

### Post-processing of the detection bounding box based on CP-Cluster

After receiving the output from the neck network, the detection head passes through a convolution layer to obtain three feature maps (*H*_1_, *H*_2,_ *H*_3_), as shown in Eq. (5).

5 $$\left\{ {\begin{array}{*{20}l} {H_{1} = {\text{Conv}}_{1 \times 1} \left[ {{\text{VoV}} \left( {FC_{2} } \right)} \right]{,80}_{H} \times {80}_{W} \times 18_{Channel} } \hfill \\ {H_{2} = {\text{Conv}}_{1 \times 1} \left[ {{\text{VoV}} \left( {FC_{3} } \right)} \right],{40}_{H} \times 4{0}_{W} \times 18_{Channel} } \hfill \\ {H_{3} = {\text{Conv}}_{1 \times 1} \left[ {{\text{VoV}} \left( {FC_{4} } \right)} \right],{20}_{H} \times {20}_{W} \times 18_{Channel} } \hfill \\ \end{array} } \right.$$

Each grid of the detection head has 18 channels. The channel dimensions are divided into three groups of prior anchor frames corresponding to different sizes in order. The six channel information pieces of each group correspond to four relative coordinates (*b*_*x*_, *b*_*y*_, *b*_*w*_, *b*_*h*_) of the prediction frame, the confidence of the prediction frame, and the number to be classified. When the coordinates of the center point of the prediction bounding box are very close to the upper-left and lower-right corners of the grid in which it is located, its offset is functionally processed and needs to be taken at negative and positive infinity, and such extreme values cannot be reached in general. In addition, too large prediction box width and height are prone to gradient explosion in the network. Therefore the sensitivity of the grid is eliminated through Eq. (6):

6 $$\begin{aligned} & b_{x} = \left( {2 \cdot \sigma \left( {t_{x} } \right) - 0.5} \right) + c_{x} ,b_{y} = \left( {2 \cdot \sigma \left( {t_{y} } \right) - 0.5} \right) + c_{y} \\ & b_{w} = P_{w} \cdot \left( {2 \cdot \sigma \left( {t_{w} } \right)} \right)^{2} ,b_{h} = P_{h} \cdot \left( {2 \cdot \sigma \left( {t_{h} } \right)} \right)^{2} \\ \end{aligned}$$

where *b*_*x*_ and *b*_*y*_ represent the coordinates of the center point of the prediction box, *t*_*x*_ and *t*_*y*_ denote the offset of the center point of the prediction box from the top-left vertex of the grid in which it is located, *c*_*x*_ and *c*_*y*_ are the horizontal and vertical distances of the top-left vertices of the grid in which the center point of the prediction box is located from the top-left vertices of the feature map, which makes the σ function values easier to take to 0 or 1 by limiting the offset range to − 0.5 to 1.5. *b*_*w*_ and *b*_*h*_ represent the width and height of the final prediction box,* P*_*w*_ and *P*_*h*_ denote the width and height of the a priori anchor box, *t*_*w*_ and *t*_*h*_ denote the offset of the width and height of the prediction box, which avoids the overgrowth of the prediction box by restricting the expansion factor to 0 to 4.

After the detection model generates all prediction boxes, it will finally start to filter them. To use the association information between different candidate boxes to obtain a better prediction box, the CP-Cluster algorithm adopts a completely different idea from the NMS^31^ algorithm, and its algorithm steps are shown in Table 2.

**Table 2:**
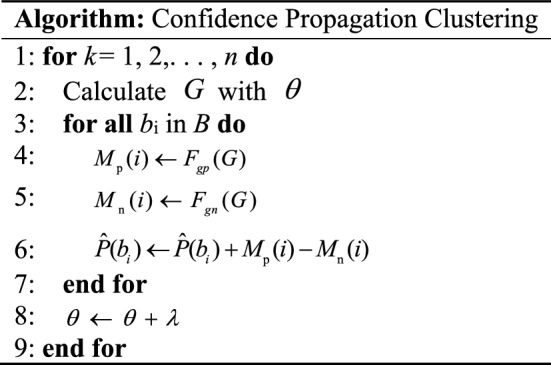
Algorithm steps for CP-Cluster.

Among them, $B = \{ b_{1} ,b_{2} , \ldots \}$ represents the set of original bounding boxes, and $\theta$ is the pre-set IoU threshold. If ${\text{IoU}} (b_{i} ,b_{j} ) > \theta$, then an undirected edge is used to connect *b*_*i*_ and *b*_*j*_, generating a set of Markov random graphs ($G = \{ g_{1} ,g_{2} , \ldots \}$). For each $g_{i} \in G$, $\varepsilon_{gi}$ is defined as its set of edges and $v_{gi}$ as defined as its set of points. When $b_{i} \in v_{gn}$, its adjacent point set $N_{bi}$ will contain all nodes connected to *b*_*i*_ in *g*_n_. Equation (7) represents the optimization objective of CP-Cluster:

7 $$\hat{P}(b_{i} ) = \hat{P}(b_{i} |N_{{b_{i} }} ,\overline{b}_{i} ) = \left\{ {\begin{array}{*{20}l} {1,} \hfill & {b_{i} \in B_{{\text{p}}} } \hfill \\ {0,} \hfill & {b_{i} \in B_{{\text{n}}} } \hfill \\ \end{array} } \right..$$

Among them, $\hat{P}(b_{i} )$ represents the confidence obtained by *b*_*i*_ after inputting information from adjacent bounding boxes and its own bounding boxes into the model. $B_{{\text{p}}}$ represents the set of true candidate boxes with the maximum overlap with the real box, and $B_{{\text{n}}}$ represents a set of redundant bounding boxes. $\overline{b}_{i}$ represents the detection confidence of the detector towards *b*_*i*_. Equation (8) provides the evaluation criteria for the weak friend set ($W_{{b_{i} }}$) and strong friend set ($S_{{b_{i} }}$). Equations (9) and (10) give the generating functions $F_{gp}$ and $F_{gn}$ of the positive and negative messages, respectively.

8 $$\left\{ {\begin{array}{*{20}l} {{\text{IoU}} (b_{j} ,b_{i} ) > \theta ,\hat{P}(b_{j} ) < \hat{P}(b_{i} ),b_{i} \in \upsilon_{gn} \to b_{j} \in W_{{b_{i} }} } \hfill \\ {{\text{IoU}} (b_{j} ,b_{i} ) > \theta ,\hat{P}(b_{j} ) > \hat{P}(b_{i} ),b_{i} \in \upsilon_{gn} \to b_{j} \in S_{{b_{i} }} } \hfill \\ \end{array} } \right.$$

9 $$M_{{\text{p}}} (i) \leftarrow \frac{Q}{Q + 1} * (1 - \hat{P}(b_{i} )) * \max \hat{P}(\hat{b}),\hat{b} \in W_{{b_{i} }}$$

10 $$\left\{ {\begin{array}{*{20}l} {\tau (b_{j} ,b_{i} ) \leftarrow \alpha * \frac{{\hat{P}(b_{j} )}}{{\hat{P}(b_{i} )}} + (1 - \alpha ) * \frac{{{\text{IoU}} (b_{j} ,b_{i} )}}{\theta }} \hfill \\ {M_{{\text{n}}} (i) \leftarrow \hat{P}(b_{i} ) * {\text{IoU}} \left( {b_{i} ,{\text{argmax}} \tau (b_{j} ,b_{i} )} \right),b_{j} \in N_{{b_{i} }} } \hfill \\ \end{array} } \right.$$

In the positive message-generation formula, the number of weak friends of bounding box *b*_*i*_ and the high confidence of weak friends are two key factors to enhance *b*_*i*_. Here, *Q* represents the number of weak friends, and $1 - \hat{P}(b_{i} )$ guarantees that the confidence of *b*_*i*_ after being enhanced is less than 1. Positive messages make it more likely that the real prediction frame will be generated from the set of candidate frames with high confidence around Morse code, reducing the interference of irrelevant signals. In the negative message-generation equation, $\tau (b_{j} ,b_{i} )$ defines the negative impact factor of negative information, which is used to select strong friend boxes that suppress *b*_*i*_. When $\alpha$ = 1, the box with the highest confidence in the strong friend set is used; when $\alpha$ = 0, the box with the most overlap with *b*_*i*_ is used. When the bounding box on one side reaches the number of inhibition times, the bounding box with the largest negative influence factor is selected to punish *b*_*i*_. The algorithm eventually outputs all prediction boxes with the same confidence, and only the prediction box with the longest horizontal dimension is reserved here to ensure the complete detection of Morse code. Finally, the Morse code detection results can be obtained by mapping the filtered prediction box information to the original time–frequency diagram according to different detection heads.

## Experiment

### Datasets

**Figure 5:**
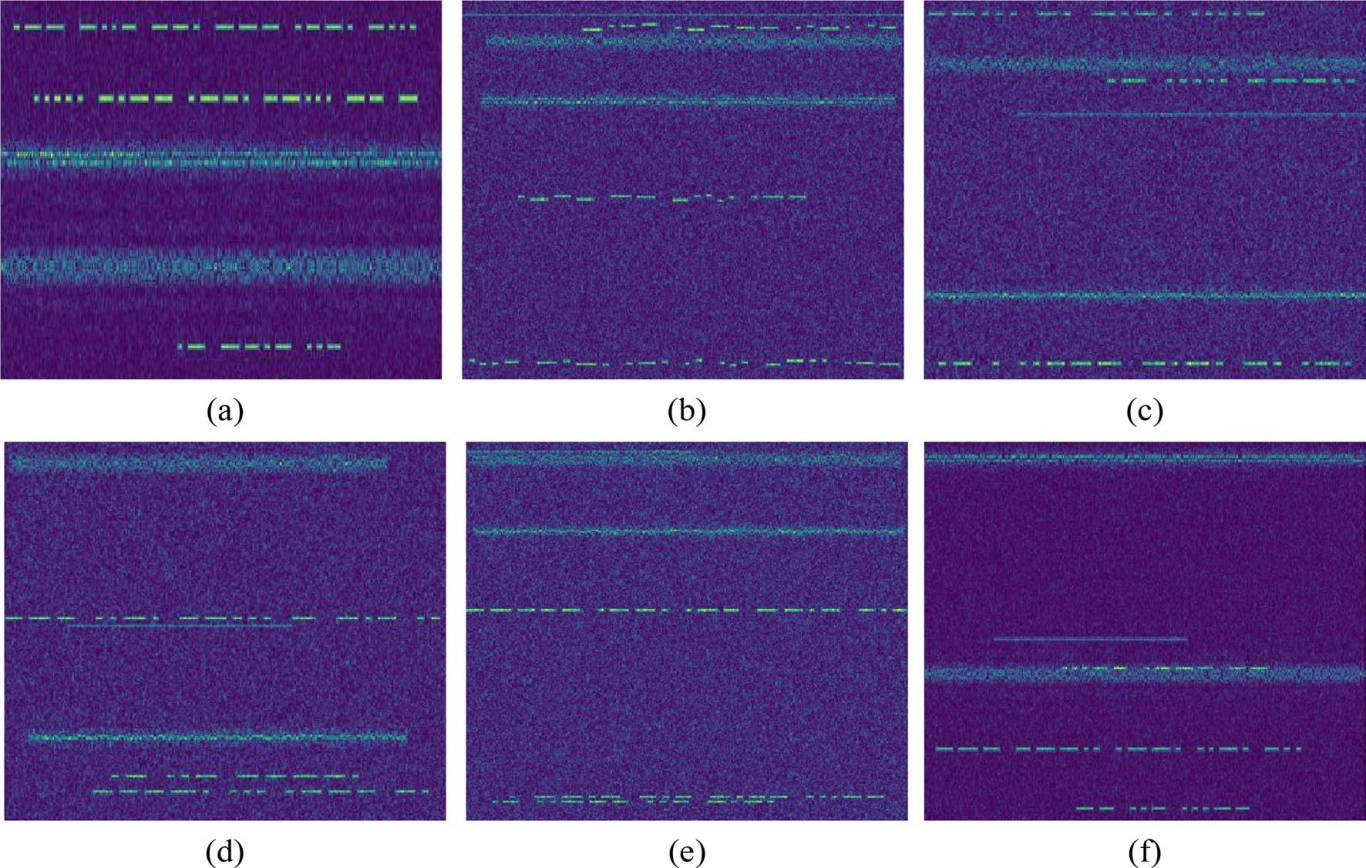
Morse code detection dataset. (**a**) General case, (**b**) presence of frequency offset, (**c**) presence of code speed deviation, (**d**) at low SNR, (**e**) frequency close to each other, (**f**) overlap with other signals.

At present, there is no open manual Morse newspaper general dataset in the Morse signal detection task, so we generated a broadband signal candidate set containing multiple Morse signals with different frequencies through simulation and other methods, and we then converted the candidate set into time–frequency images through short-time Fourier transform. The quality of shortwave communication is easily affected by ionospheric changes, and unstable channels may also cause frequency drift of Morse signals. Generally, Morse's communication frequency band contains various modulation signals. We selected representative FSK, PSK, and AM modulation methods to modulate baseband signal sequences or actually collected human voice and music signals to the vicinity of Morse's signals, and we enriched the background independent signals based on white noise interference. The dataset was also used to simulate frequency drift and code rate fluctuation, and we added -5 to 0 dB random white noise to the background. The training set contained 2072 pictures in total, and the verification set contained 350 pictures. Each time–frequency diagram contained at least one Morse signal. The center frequency of the Morse signal was within 5 ~ 12 MHz, the frequency bandwidth was 2 MHz, and the duration of different signals was within 3 ~ 8 s. Figure 5 shows an example of the datasets.

### Experimental setup and data enhancement

We used a Windows 10 system, and the program code compilation and running environment was Python 3.8, pythoch1.10.0, and cuda11.3. All models were run on NVIDIA RTX3080Ti GPU. The specific parameter settings of the experiment are shown in Table 3.

**Table 3:** Experimental parameter settings.

Parameters	Value	Parameters	Value
Image size	640*640	epochs	100
Momentum	0.937	batch-size	16
Weight_decay	0.0005	conf-thres	0.01
Warmup_epochs	3.0	iou-thres	0.5

To enhance the generalization of the detection model and avoid the decline in model detection performance due to the difference in color characteristics between the training set and the test set, we randomly selected 90% of the training time–frequency images for graying processing before each round of training, making the detection model more inclined to learn the geometric characteristics of Morse code than the color characteristics. To enhance the robustness of the model, the mosaic strategy was used to enhance the data; In addition, we pre-clustered a suitable priori anchor box according to the real boxes in the dataset. The specific size is shown in Table 4.

**Table 4:** Priors’ anchor size.

Priors’ anchor size		
209 × 9	404 × 31	556 × 18
349 × 11	430 × 7	580 × 26
353 × 20	524 × 10	586 × 35

### Performance evaluation index

The indicators to measure the performance of the detection model in this paper were Precision, Recall, AP0.5, AP0.5:0.95, F2 score, Parameters, giga floating-point operations per second (GFLOPs), and frames per second (FPS). Precision and Recall represent the precision and recall of the detection model, respectively. Because there is a negative correlation between the two, the value of the area under the curve was used to evaluate the overall detection quality of the model. The setting of IoU threshold also affected the performance of AP. The higher the IoU threshold, the stricter the evaluation criteria. Considering that the purpose of Morse code detection is to provide a data basis for later decoding work, the missing detection of Morse signal is more serious than error detection, so the F2 score was selected to increase the influence weight of Recall in performance evaluation, and its calculation formula is as shown in Eq. (11). Parameters and GFLOPs were used to measure the parameters and computational complexity of the model. FPS was used to measure the overall processing speed of the model, and the total time spent from inputting signal data into the model to outputting the detection results was the total time spent.

11 $$F_{\beta } = \frac{{(1 + \beta^{2} ) \times precision \times recall}}{{(\beta^{2} \times precision) + recall}},\beta = 2$$

### Experimental results and analysis

#### Model selection

The experiments used the representative Faster-RCNN, SSD, and YOLOv8s in the target detection algorithm to detect multiple Morse codes. After 100 cycles of training, the performance evaluation of each detection model was done, and the results are shown in Table 5.

**Table 5:** Comparison of different models.

Models	Memory (MB)	AP0.5	AP0.5: 0.95
SSD	90.6	0.936	0.584
Faster-RCNN	314.2	0.994	0.667
YOLOv5s	13.7	0.993	0.665
YOLOv8s	85.4	0.992	0.673

Among them, the AP0.5 of SSD was only 0.936 and was prone to incomplete detection. Although Faster-RCNN had good detection performance, its processing speed and memory usage could not meet the practical requirements. YOLOv8s and YOLOv5s detectors gave better consideration to detection accuracy and reasoning speed. The AP0.5:0.95 of YOLOv8s was the highest, 0.673, but YOLOv5s performed better when IoU = 0.5. In addition, the results of 10 control experiments show that the FPS of YOLOv5s was generally approximately 9% higher than that of YOLOv8s, and that YOLOv5s had fewer parameters and less computational complexity. Therefore, we chose to design our Morse code detection algorithm based on YOLOv5s.

#### Ablation test

To verify the function of each module in YFDM, we conducted four groups of experiments to ablate each module. The first group was the original YOLOv5s; the second group used the DCN and C3 modules to reconstruct the backbone network of YOLOv5s; the third group used the GSConv and VOV-GSCSP modules to reconstruct the neck network of YOLOv5s; and the fourth group combined the second group and the third group, namely the YFDM detection model. Under the same training conditions, the experiment was repeated 10 times for each group. The experimental results are shown in Tables 6 and 7. Compared with the first group of experiments, the AP0.5:0.95 of the second group increased by 0.5%, the F2 score increased by 0.5%, and GFLOPs decreased by 19.6%. Although the DCNv2 module slightly increased the number of model parameters, it improved the detection accuracy and reduced the computational complexity. In the third group, the number of model parameters and GFLOPs decreased by 16.8% and 19.7%, respectively. Although AP0.5:0.95 showed slight losses, compared with the optimization effect of model parameters and computational complexity, these losses were acceptable. The fourth group of experimental results show that the proposed model obtained the highest F2 score and AP0.5:0.95, and that its model parameters and GFLOPS were only 84.9% and 61.1% of those of YOLOv5s.

**Table 6:** Results of ablation experiment.

Group	Precision	Recall	F2	AP0.5:0.95	Parameters (M)	GFLOPs
1	0.992	0.987	0.988	0.665	7.022	15.94
2	0.993	0.993	0.993	0.670	7.138	12.88
3	0.991	0.994	0.993	0.664	5.845	12.80
4	0.989	0.995	0.994	0.670	5.961	9.74

**Table 7:** Parameters and GFLOPs of the proposed model (+ / − represents the change compared with YOLOv5s).

	Layer	Params	GFLOPs		Layer	Params	GFLOPs
1	Conv	3520	0.73	14	VoVGSCSP × 3	219,328	0.46 (− 0.7)
2	DCNv2	26,299 (+ 7739)	0.4 (− 0.56)	15	GSConv	18,240 (− 14,784)	0.06 (− 0.05)
3	C3	18,816	0.98	16	Upsample	0	0
4	DCNv2	89,435 (+ 15,451)	0.2 (− 0.75)	17	Concat	0	0
5	C3	115,712	1.49	18	VoVGSCSP × 3	56,416 (− 34,464)	0.48 (− 0.69)
6	DCNv2	326,299 (+ 30,875)	0.1 (− 0.85)	19	GSConv	75,584 (− 72,128)	0.24 (− 0.23)
7	C3	625,152	2.01	20	Concat	0	0
8	DCNv2	1,242,395 (+ 61,723)	0.05 (− 0.9)	21	VoVGSCSP × 3	186,560 (− 109,888)	0.36
9	C3	1,182,720	0.95	22	GSConv	298,624 (− 291,712)	0.24 (− 0.23)
10	SPPF	656,896	0.53	23	Concat	0	0
11	GSConv	69,248 (+ 62,336)	0.06 (− 0.05)	24	VoVGSCSP × 3	733,568 (− 449,152)	0.35 (− 0.6)
12	Upsample	0	0	25	Detect	16,182	0.05
13	Concat	0	0		Total	5,960,994 (− 1,061,332)	9.74 (− 6.2)

Table 8 compares the computational complexity and number of model parameters between the suggested model and different YOLO versions. The data indicates that compared to other YOLO variants in the same class, YOLOv5s and YOLOv8s offer greater lightweight benefits. In addition, the improved YFDM model using YOLOv5s as the base model in the experiments in “Model selection” section has fewer model parameters and lower computational complexity, which provides favorable conditions for the deployment of the algorithm on the mobile device side. This is because there is no discernible difference in the detection performance between the two in the Morse code detection task.

**Table 8:** Comparison of parameters and GFLOPs for different YOLO versions.

YOLO version	Image size	Parameters (M)	GFLOPs
YOLOv3	640 × 640	61.53	193.89
YOLOv4	640 × 640	52.50	119.83
YOLOv5s	640 × 640	7.022	15.94
YOLOv6s	640 × 640	17.19	44.12
YOLOv7	640 × 640	36.49	103.50
YOLOv8s	640 × 640	11.20	28.60
Our method	640 × 640	5.961	9.74

#### Selection of boundary box regression loss function

To test the impact of different IoU loss on the detection performance, we selected CIoU loss, DIoU loss^32^, EIoU loss^33^, SIoU loss^34^, and WIoU loss^35^ to train the proposed model. Table 9 shows the test results. CIoU loss and DIoU loss had the highest F2 score, followed by WIoUv1 loss, and SIoU loss had the lowest score. The highest AP0.5:0.95 of 0.673 was obtained for WIoUv1 Loss, followed by 0.67 for CIoU loss, and the AP0.5:0.95 of other IoU loss was lower than 0.67. DIoU loss had the highest FPS, WIoUv1 loss was second only to DIoU loss, and SIoU loss and EIoU loss had relatively close FPS values. CIoU loss had the lowest FPS, 102.044.

**Table 9:** Impact of different IoUs on detection accuracy.

IoU Loss	Precision	Recall	F2	AP0.5:0.95	FPS
CIoU	0.989	0.995	0.9938	0.670	102.04
DIoU	0.992	0.995	0.9944	0.663	105.73
EIoU	0.988	0.992	0.9912	0.668	103.33
SIoU	0.994	0.986	0.9876	0.668	103.40
WIoUv1	0.991	0.993	0.9926	0.673	104.26

The DIoU loss uses the Euclidean distance of the center points of the two frames for regression, and it had the highest FPS. However, owing to the lack of specific coordinate information of Morse code, even though the prior frame size had been determined, the AP0.5:0.95 of the DIoU loss was still the lowest, 0.663. CIoU loss considers the overlapping area, center point distance, and aspect ratio of prediction frames. EIoU loss calculates the length and width loss of real frames and prediction frames on the basis of CIoU loss. Because the inverse trigonometric function calculation involved in CIoU loss affects the reasoning speed, the FPS of EIoU loss was slightly faster, but the detection accuracy and F2 score of the two were not significantly different. SIoU loss takes the vector angle between two frames into account, and its precision was the highest, 0.994, but the F2 score was the lowest, 0.9876, so there was a relatively high probability of missing detection. WIOUv1 loss constructs attention-based bounding-box loss, which can significantly weaken the punishment of geometric metrics for low-quality examples. The proposed model achieved the highest AP0.5:0.95 and a relatively high FPS.

#### Impact of CP-Cluster on detection performance

**Figure 6:**
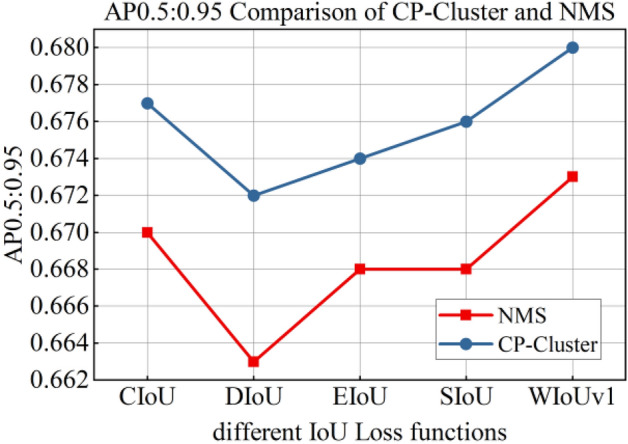
AP0.5:0.95 of CP-Cluster and NMS.

**Figure 7:**
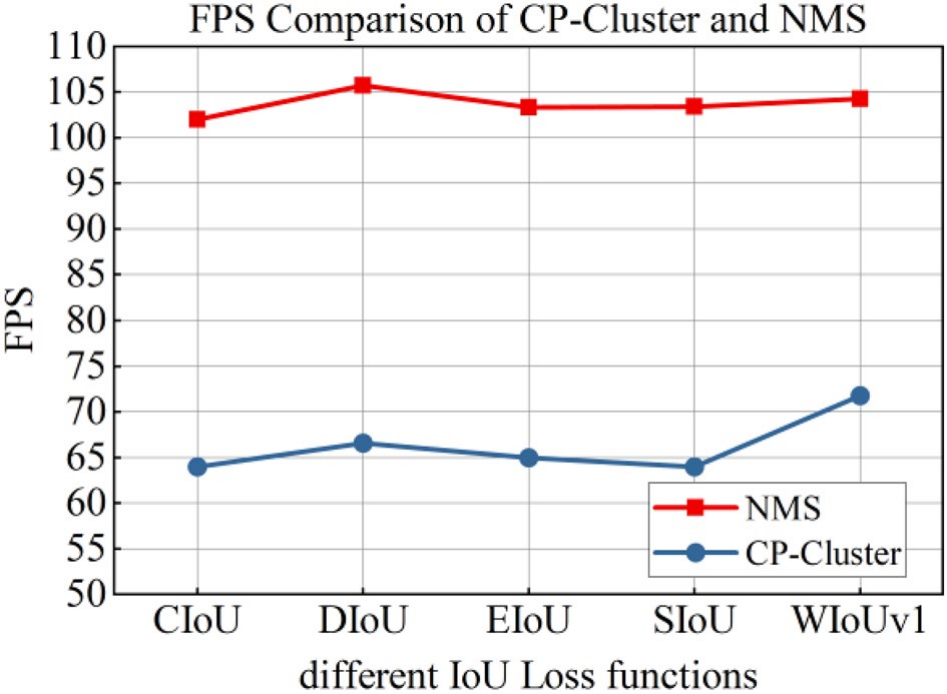
FPS of CP-Cluster and NMS.

In this experiment, the training model in “Selection of boundary box regression loss function” section was used for the reasoning prediction box, and NMS and CP-Cluster algorithms were used for candidate box screening. The processing results of the two algorithms are shown in Figs. 6 and 7.

It can be seen from the figure that the AP0.5:0.95 of the CP-Cluster method was significantly improved compared with the NMS method. When WIoUv1 was selected as the loss function of the bounding box, the AP0.5:0.95 of this model reached 0.68, which was 2.26% higher than the first group of models in the ablation experiment in “Ablation test” section. The CP-Cluster method requires a certain amount of computing power for message propagation, which increases the overall time of model output results. However, its FPS could still reach 71.76, which can meet the real-time requirements of Morse code detection.

#### Final detection effect of the proposed model

**Figure 8:**
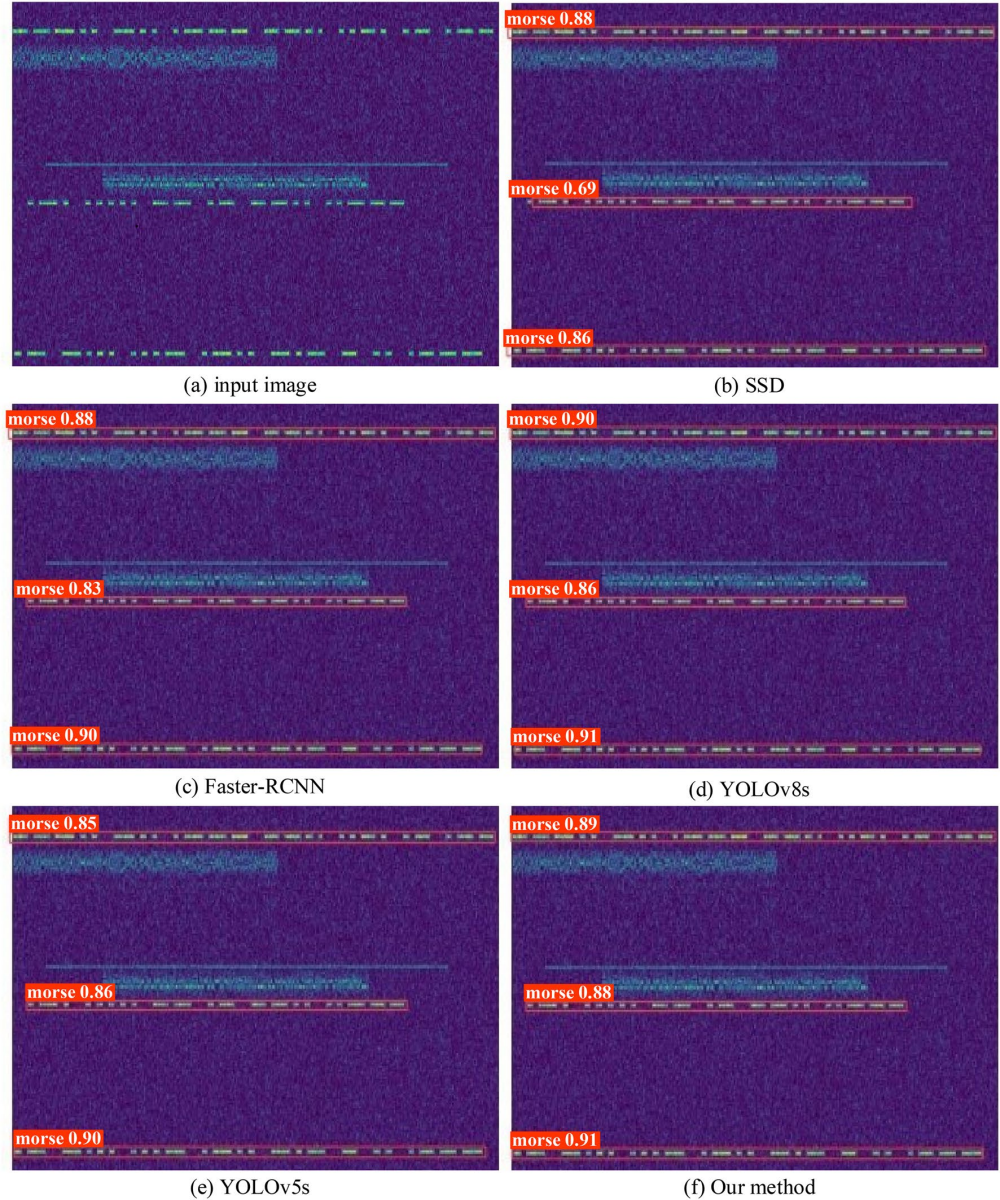
Results of different detection models.

Figure 8 shows the detection effects of the proposed model and other models. The proposed model is trained by utilizing WIoUv1 as a loss function and, during the post-processing phase, employing the CP-Cluster technique to filter the anticipated bounding boxes. When the SSD detector was used, one of the Morse code was not completely detected. The detection results of Faster-RCNN and YOLOv5s are closer to each other, but Faster-RCNN had difficulty meeting the real-time requirements of Morse code detection and required a large amount of memory space of the hardware device. The proposed model had a similar detection effect as YOLOv8s, and their detection frames could fit the Morse message region more accurately, but the proposed model has the advantages of a light weight and processing delay, and it is more suitable for Morse code detection task.

## Conclusion

Based on the embeddability of Morse code detection algorithm, we designed a Morse code detection algorithm called YFDM combining the advantages of DCNV2, GSConv, and VOV-GSCSP modules. In the training phase, we analyzed the impact of different IoU loss functions on detection performance, and we found that when WIoUv1 was used as the loss function of the bounding box, the training model could achieve the highest AP0.5:0.95 and a relatively fast processing speed. In the candidate box processing phase, although the introduction of CP-Cluster method increased the overall time consumption, it significantly improved the detection quality. Finally, the YFDM algorithm effectively reduced the number of parameters and the computational complexity of the algorithm model on the premise of ensuring sufficient detection accuracy and low processing delay.

## Data Availability

The datasets used and/or analysed during the current study available from the corresponding author on reasonable request.
